# Oxidative Stress-Induced Cellular Senescence in Aging Retina and Age-Related Macular Degeneration

**DOI:** 10.3390/antiox11112189

**Published:** 2022-11-05

**Authors:** Ryo Terao, Tazbir Ahmed, Ayana Suzumura, Hiroko Terasaki

**Affiliations:** 1 Department of Ophthalmology, Graduate School of Medicine, The University of Tokyo, Tokyo 113-8654, Japan; 2 Department of Ophthalmology & Visual Sciences, Washington University School of Medicine in St. Louis, St. Louis, MO 63110, USA; 3 Department of Ophthalmology, Graduate School of Medicine, Nagoya University, Nagoya 466-8550, Japan; 4 Institutes of Innovation for Future Society, Nagoya University, Nagoya 464-8601, Japan

**Keywords:** aging, age-related macular degeneration, cellular senescence, inflammation, oxidative stress

## Abstract

Aging leads to a gradual decline of function in multiple organs. Cataract, glaucoma, diabetic retinopathy, and age-related macular degeneration (AMD) are age-related ocular diseases. Because their pathogenesis is unclear, it is challenging to combat age-related diseases. Cellular senescence is a cellular response characterized by cell cycle arrest. Cellular senescence is an important contributor to aging and age-related diseases through the alteration of cellular function and the secretion of senescence-associated secretory phenotypes. As a driver of stress-induced premature senescence, oxidative stress triggers cellular senescence and age-related diseases by inducing senescence markers via reactive oxygen species and mitochondrial dysfunction. In this review, we focused on the mechanism of oxidative stress-induced senescence in retinal cells and its role in the pathogenesis of AMD.

## 1. Introduction

Aging is an inevitable and irreversible process characterized by deterioration at the cell, organelle, tissue, and organ levels [1]. It is the primary risk factor for several cardiovascular, neurodegenerative, immunological, musculoskeletal, and metabolic diseases [2]. As life expectancy has increased, it is essential to develop prophylactics and therapeutics for age-related diseases.

Age-related macular degeneration (AMD) is the most common retinal disease caused by aging and is the leading cause of blindness in individuals over 60 years old [3]. The projected number of patients with early- and late-stage AMD is increasing globally [4]. AMD impairs the central visual field by affecting the macula, the central part of the retina. In the early stage, patients with AMD present with drusen, a lipoprotein-rich material in the sub-retinal pigment epithelium (RPE) or beneath the neurosensory retinal space [5]. In the advanced stage, there are two types of AMD—dry and wet AMD [6]. Wet AMD is characterized by choroidal neovascularization, which causes subretinal hemorrhage, macular edema, and intravitreous hemorrhage. Dry AMD is a slowly progressive retinal degeneration caused by irreversible photoreceptor death. Although these two phenotypes have different clinical manifestations, both can lead to severe vision loss. Although AMD is caused by complex interactions among genetic factors associated with lipid metabolism, the inflammasome, the immune response, and environmental factors including dietary intake, smoking, obesity, and light exposure, aging and age-associated factors are the primary drivers of AMD [7]. Because its molecular pathogenesis is unknown, an understanding of the pathological mechanism would facilitate the development of therapeutics for early and late AMD. 

Oxidative stress is a key factor in AMD pathology, aging, and age-related diseases [8]. It induces damage to cells and organelles, leading to pathological signaling [9]. The resulting continuous and cumulative oxidative stress in retinal tissue triggers AMD [10]. As a cellular response to oxidative stress, cellular senescence is an important contributor to aging and age-related diseases [11]. It is characterized by cell cycle and proliferation arrest [12]. Given that the signaling network between oxidative stress and cellular senescence contributes to the development of age-related diseases, oxidative stress is likely to trigger AMD by inducing cellular senescence. Therefore, in this review, we focused on the relationship between oxidative stress and cellular senescence and their effects on age-related diseases of the retina.

## 2. Oxidative Stress and Cellular Dysfunction in the Eye

**Figure 1:**
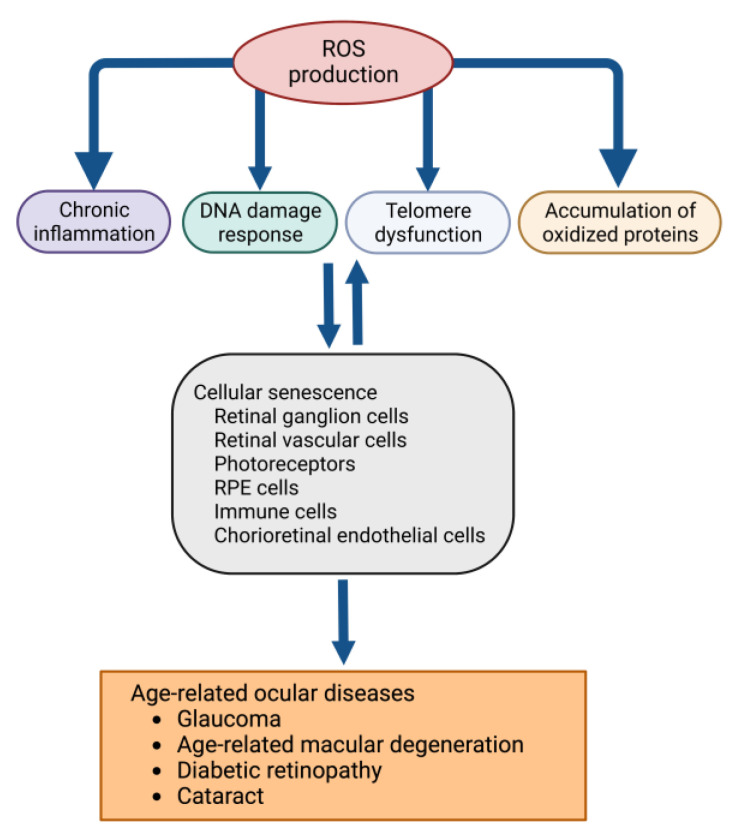
Biological roles of oxidative stress in cellular senescence in the retina.

The development of age-related diseases is affected by numerous genetic and environmental factors. Oxidative stress is a strong driver of age-related diseases irrespective of the genetic and environmental factors [13]. Oxidative stress occurs from an imbalance between the production of reactive oxygen species (ROS) and antioxidant defenses [14]. In aging, antioxidants deteriorate, causing cellular and mitochondrial dysfunction by elevating intracellular ROS. ROS are mainly produced in mitochondria, an organelle essential for the synthesis of ATP (adenosine triphosphate) [15]. Excess ROS leads to cellular dysfunction and pathologic conditions in various organs. ROS oxidize biological macromolecules such as proteins, lipids, and nucleic acids (Figure 1) [16]. For instance, oxidative DNA damage results in telomere shortening, DNA methylation, histone deacetylation, and mitochondrial dysfunction, inducing transcriptomic changes associated with the aging process [17,18]. ROS also triggers the accumulation of ubiquitinated proteins by downregulating the 26S proteasome. The resulting insufficient protein degradation leads to intracellular aggregation of the oxidized proteins [19]. 

Mitochondrial DNA (mtDNA) plays a pivotal role in oxidative stress-induced cellular dysfunction. ROS generated in mitochondria damages mtDNA and other mitochondrial constituents. ROS oxidize mtDNA, which leads to mitochondrial dysfunction [20]. Conversely, damaged mtDNA facilitates ROS production, resulting in mitochondrial dysfunction and the production of yet-more ROS. Via a positive feedback loop, oxidative stress and mitochondrial dysfunction promote an inflammatory response via nuclear factor-κB (NF-κB) signaling by activating the NLRP3 inflammasome and cGAS/STING pathway, ultimately resulting in chronic inflammation and age-related diseases [21,22].

The balance between ROS and antioxidants is maintained by multiple pathways. NF-κB is a major signaling factor that responds to ROS. In mammals, NF-κB consists of five proteins—p50, p52, p65/RelA, c-Rel, and RelB. ROS activates the multi-subunit IκB kinase complex, which phosphorylates IκB [23], leading to its ubiquitin-dependent degradation. Activated NF-κB translocates into the nucleus and activates target genes [24]. By upregulating interleukin (IL)-6, IL-8, tumor necrosis factor (TNF)-α, monocyte chemoattractant protein (MCP)-1, C-X-C motif ligand (CXCL)1, and intercellular adhesion molecule (ICAM-1), NF-κB promotes inflammation and angiogenesis. 

A member of the basic leucine zipper transcription factor family, nuclear factor-erythroid 2-related factor 2 (Nrf2) is a transactivator of multiple antioxidants [25]. Nrf2 maintains redox homeostasis by interacting with antioxidant response element (ARE). In the homeostatic state, Nrf2 is ubiquitinated by Kelch-like ECH-associated protein 1 (Keap1), a negative regulator of Nrf2. Under oxidative stress, conformational changes in Keap1 stabilize Nrf2 and dissociate it from Keap1 [26]. As a result, Nrf2 binds to ARE, regulating the expression of antioxidant and anti-inflammatory genes including glutathione peroxidase, glutathione reductase, superoxide dismutase, heme oxygenase-1, thioredoxin reductase, ferritin, and NAD(P)H quinone oxidoreductase 1 [27]. Through its transcriptomic activity, Nrf2 reduces the ROS levels and oxidative stress [28]. However, Nrf2 activity decreases with age, enhancing the susceptibility to oxidative stress [29]. Therefore, chronic oxidative stress is implicated in aging-related diseases. 

There are numerous studies that have investigated the biological roles of oxidative stress on the pathogenesis of AMD. Among the retinal cells, RPE cells are particularly associated with AMD pathobiology, and RPE cell dysfunctions by oxidative stress plays a pivotal role in the development of AMD. RPE cells promote retinal homeostasis through the blood–retina barrier (BRB), phagocytizing photoreceptor outer segments, and regulating the retinoid cycle. Because aging degrades RPE function, it disrupts the BRB and causes photoreceptor dysfunction. AMD patients exhibited decreased autophagosome and autophagic markers, indicating that dysregulated autophagy in RPE cells leads to AMD [30]. Therefore, insufficient autophagy in RPE cells causes ubiquitinated protein aggregation and the accumulation of drusen [31]. These effects impair the RPE and trigger the development of AMD. Oxidative stress by hydrogen peroxide induces mitochondrial DNA damage and cell death [32,33,34], which triggers RPE cell dysfunctions. The targeted deletion of *Sod2* encoding manganese superoxide dismutase in RPE cells leads to increased oxidative stress and dysfunctions in RPE cells and photoreceptors [35]. Dysfunctional RPE cells by oxidative stress drives inflammation. ROS in RPE cells promote NF-κB signaling and nod-like receptor family pyrin domain containing 3 (NLRP3) inflammasome [36,37], which further upregulates IL-1β and IL-18 through the activation of caspase-1 [38]. ROS is also associated with angiogenesis in the retina and choroid [39]. Briefly, ROS induces VEGF expression in vascular endothelial cells, vascular smooth muscle cells, and macrophages. Mechanistically, angiogenic response is mediated through hypoxia inducible factor 1α (HIF-1α) [40]. Furthermore, VEGF induces ROS production and activates NADPH oxidase in choroidal endothelial cells. The inhibition of NADPH oxidase suppresses a murine laser induced choroidal neovascularization model [41], suggesting the close relationship between oxidative stress and choroidal angiogenesis.

## 3. Molecular Biological Relationships between Oxidative Stress and Cellular Senescence 

Cellular senescence is the state of permanent cell cycle arrest, initially reported as a limited proliferative potential of normal cells in culture [42]. Cellular senescence is caused by various stressors such as oxidative stress, DNA damage, organelle stress, telomere dysfunction, and aging [43]. Autophagy dysfunction, metabolic disturbance, abnormal inflammatory response, and growth factors are important drivers of cellular senescence. Cellular senescence was formerly considered an intrinsic programmed response to adaption and is essential for tumor suppression. Cellular senescence is also involved in maintaining physiological homeostasis in wound repair, remodeling, and survival. The accumulation of senescent cells triggers various age-related diseases such as Parkinson disease, Alzheimer disease, pulmonary fibrosis, osteoarthritis, atherosclerosis, and age-related ocular diseases (glaucoma and cataract) via a variety of molecular mechanisms [44]. Senescent cells have an altered structure and organelle morphology and functionality. Senescence causes cells to become enlarged, flat, multivacuolated, and multinucleated [45]. Mitochondria and lysosomes control cellular senescence. Senescent cells exhibit impaired lysosomal digestion, resulting in the accumulation of cellular garbage including protein aggregates and lipofuscin [46]. Lysosome dysfunction impairs mitochondrial turnover. The result is functionally deficient mitochondria, and increased ROS production and oxidative stress, enhancing the synthesis of lipofuscin in a feedback loop [47]. Lipofuscin is produced by senescent cells and its accumulation is a hallmark of cellular senescence [48,49,50]. Lipofuscin is an aggregate of lipids, metals, and misfolded proteins, which constitute a lipoprotein-rich material known as drusen. Lipofuscin accumulates in the lysosomes of senescent cells. During aging, DNA damage promotes intracellular lipofuscin accumulation [51]. Autophagy dysfunction leads to the accumulation of lipofuscin in lysosomes. Intracellular lipofuscin, together with other senescence markers such as SA β-Gal, accumulates in senescent cells of various types [49,52]. As a manifestation of cellular senescence, oxidative stress promotes the synthesis of lipofuscin. Senescent cells with intracellular lipofuscin show higher ROS production and suppressed antioxidant defenses [53]. The inhibition of mitochondrial fission promotes lipofuscinogenesis, and a mitochondrion-targeted antioxidant (mitoTEMPO) inhibited the accumulation of lipofuscin in human fibroblasts and HeLa cells [52]. 

**Figure 2:**
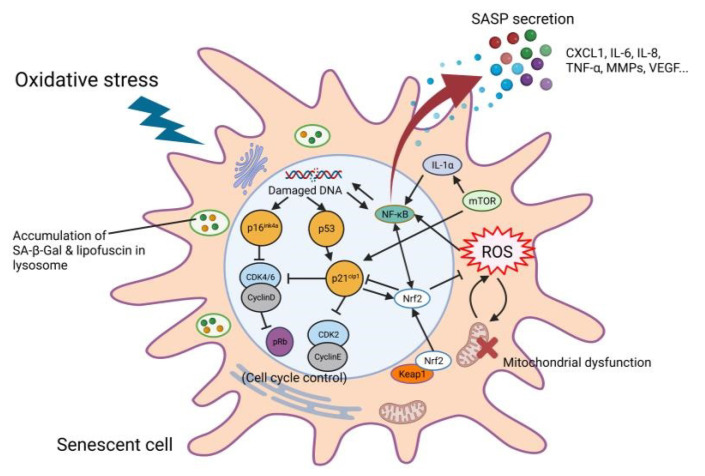
Schematic representation and molecular signaling network of a senescent cell.

There are three types of cellular senescence—replicative senescence, stress-induced premature senescence (SIPS), and developmentally programmed senescence (DPS) [54]. In replicative senescence, the ability of somatic cells to divide diminishes due to repeated cellular replication and telomere shortening [55]. SIPS is induced by various stressors (e.g., ultraviolet radiation, oxidative stress, and oncogene activity) [56]. DPS has developmental and morphogenetic functions during embryonic development and has been proposed as the evolutionary origin of senescence [57]. Oxidative stress triggers SIPS in various cell types, considering that sustained oxidative stress induces cellular senescence and antioxidants suppress cellular senescence (Figure 2) [11,58]. Hydrogen peroxide promotes cellular senescence in vascular endothelial cells and fibroblasts [58,59]. In turn, senescent cells produce high levels of ROS and have increased oxidative DNA damage [11,60], suggesting a close relationship between oxidative stress and cellular senescence. 

Stressors such as DNA damage, telomere erosion, and oxidative stress trigger cellular senescence by acting on the p16^INk4a^/Rb and p53/p21^Cip1^ pathway, leading to cell cycle arrest. p16^INK4a^ and p21^Cip1^ are cyclin-dependent kinase (CDK) inhibitors. Inhibition of CDK arrests cell cycle progression from the G_1_ to S phase, preventing DNA replication [61]. They were discovered as tumor suppressors in tumors from various tissues, and loss of p53, p16^INK4a^, or p19^ARF^ leads to the inactivation of cellular senescence and malignant transformation [62]. p16^INK4a^ and p21^Cip1^ trigger general aging, cellular senescence, and age-related disorders [63]. p16^INK4a^ is encoded by CDK inhibitor 2A in the INK4a/ARF locus on chromosome 9p21.3, which also encodes p19^ARF^ [64]. p16^INK4a^ binds to CDK4/6 and inhibits CDK4/6-cyclin D complex formation, decelerating the cell cycle by inhibiting S phase by preventing the phosphorylation of hypophosphorylated retinoblastoma protein (Rb) (p16^INK4a^/pRb pathway). Via these mechanisms, p16^INK4a^ suppresses tumor progression, and the loss of p16^INK4a^ leads to malignancy and/or a higher grade of malignancy [65]. In addition, p16^INK4a^ is associated with aging, apoptosis, cell invasion, and angiogenesis. p16^INK4a^ and p19^ARF^ increase in the aged brain, heart, and lung tissues, implicating p16^INK4a^ and p19^ARF^ in physiological aging and the deterioration of organ function with age [66,67]. Moreover, selective elimination of p16^INK4a^ positive cells extends the lifespan, suppresses tumorigenesis, and prevents age-related disorders including cataract [68]. Therefore, cellular senescence associated with the p16^INK4a^/Rb pathway is a critical driver of age-related diseases, and therapeutic removal of senescent cells (senolysis) could prevent age-related diseases.

p21^Cip1^ is a cyclin-dependent kinase inhibitor encoded by CDK inhibitor 1A on chromosome 6p21.2 [69]. p21^Cip1^ acts downstream of p53 (encoded by TP53). ROS activate the p53/p21^Cip1^ pathway by triggering the DNA damage response. Upon DNA damage or endogenous/exogenous stressors, p53 is activated and transcriptionally upregulates target genes including p21^Cip1^ (p53/p21^Cip1^ pathway) [70]. p21^Cip1^ binds to CDK2 and CDK4/6, causing cell cycle arrest at the G1 and S phases [71]. p21^Cip1^ contributes to DNA repair, the modulation of apoptosis, and transcriptional regulation as well as cell cycle arrest [72].

Senescent cells have increased senescence-associated β-galactosidase (SA β-Gal) activity. The evaluation of SA β-Gal activity is used to identify senescent cells. Cells with replicative senescence or SIPS show β-galactosidase activity at pH 6.0, whereas normal cells have β-galactosidase activity at pH 4.5 [73].

Oxidative stress upregulates senescence markers (Figure 2). Although the abundance of senescence markers differs among tissues, p16^Ink4a^, p21^Cip1^, and SA β-Gal are activated by oxidative stress. For instance, p16^Ink4a^, p53/p21^Cip1^, and SA β-Gal activities are increased in arterial endothelial, smooth muscle, and immune cells by oxidative stress, triggering cardiovascular diseases [74]. Human peritoneal mesothelial cells show increased expression of early population doubling level cDNA-1, p16^Ink4a^, and SA β-Gal activity with increasing passage number, together with elevated ROS levels and reduced proliferation [75]. The activity of SA β-Gal, the expression of p53, and nuclear γH2AX foci are increased in disc cells by hydrogen peroxide [76]. In human dental pulp cells, hydrogen peroxide activates SA β-Gal activity, the p53/p21^Cip1^ pathway, and the secretion of several inflammatory cytokines; collectively, these responses are termed the senescence-associated secretory phenotype (SASP) [77].

Senescent cells secrete proinflammatory cytokines and growth factors such as IL-6, IL-8, CXCL1, CXCL2, CSF-1, transforming growth factor (TGF)-β, matrix metalloproteinases (MMPs), and vascular endothelial growth factor (VEGF) [78]. SASPs induce immune cell recruitment and inflammation in an autocrine and/or paracrine manner. Additionally, SASPs from senescent cells induce senescence in healthy cells [79]. Consequently, SASPs ultimately promote parasenescence and chronic inflammation, leading to chronic inflammation. SASPs are mediated by p38 mitogen-activated protein kinase, NF-κB, Notch, and mammalian target of rapamycin (mTOR) signaling. NF-κB signaling is a primary inducer of SASPs by oxidative stress [80]. Oncoprotein H-Ras V12 activates NF-κB signaling and induces senescence in IMR-90 fibroblasts, upregulating inflammatory cytokines such as IL-6, IL-8, CXCL1, and ICAM-1 in a p65-dependent manner [81]. In terms of the relationship between SASP and oxidative stress, Han et al. found that hydrogen peroxide promoted the expression of suppressor of cytokine signaling 3, IL-1α, IL-1β, IL-6, IL-8, and C–C motif chemokine ligand3 as well as senescence markers. The effect was suppressed by STAT3 or NF-κB inhibitor [79]. Moreover, the suppression of NF-κB signaling bypasses senescence induced by the p53/p21^Cip1^ pathway [82]. These findings implicate NF-κB signaling in promoting senescence and the SASP. 

Studies regarding the biological roles of Nrf2 in senescence have focused on its anti-senescent effects. The expression and transcriptional activity of Nrf2 decline during aging and Nrf2 contribute to lifespan extension [83,84,85,86]. Senescent cells exhibit a lower expression of Nrf2, and Nrf2 silencing increases p16^Ink4a^ expression and SA β-Gal activity [87]. Genetic suppression of Keap1 decreased the senescence markers (p16^Ink4a^ and p21^Cip1^) and SASPs (IL-1 β, IL-6, and TNF-α) in old mice, and attenuated the aging phenotype in the salivary glands [88]. Moreover, Nrf2 knockout promoted the expression of senescence markers and SASP, aggravating inflammation in the hippocampus [89]. In turn, p21^Cip1^ inhibits the degradation of Nrf2. Under oxidative stress, p21 binds to Nrf2 by interacting with ^9^DLG and ^79^ETGE motifs, stabilizing Nrf2 and activating signaling [90]. As a result, the p21^Cip1^-Nrf2 axis contributes to neuroprotection and survival [91]. Although the biological interactions are unclear, there is a close molecular relationship between cellular senescence and the KEAP1-Nrf2 system [92]. 

mTOR also regulates the interaction between oxidative stress and cellular senescence. As a critical regulator of immunity, mTOR promotes the innate inflammatory response by regulating cytokines and chemokines [93]. mTOR signaling is involved in aging and age-related diseases via complex molecular interactions [94]. The inhibition of mTOR extends the lifespan by delaying age-related diseases and improving physical function [95,96]. mTOR upregulates inflammatory SASPs including IL-6, IL-8, and CXCL1. The production of SASPs is regulated by translation of membrane-bound cytokine IL-1α, increasing NF-κB activity [97]. mTOR inhibitors ameliorate senescence in immune cells [98]. Rapamycin suppresses oxidative stress-induced senescence markers (p16^Ink4a^, p21^Cip1^, and SA β-Gal) and SASPs (IL-6, TNF-α, CXCL1, MMP3, CCL9, and MCP2). The effects of rapamycin on senescence are complex, and in part mediated by Nrf2. However, the mechanism is apparently the p16^Ink4a^-independent pathway [99]. 

## 4. Roles of Oxidative Stress-Induced Cellular Senescence in Retina and Age-Related Macular Degeneration

Senescence markers including p16^INK4a^ and p19^ARF^, and p21^Cip1^ are essential for fetal ocular development [100]. Taspase-1 knockout mice showed microphthalmia or anophthalmia as well as craniofacial anomalies. In this model, p16^INK4a^ and p19^ARF^ were upregulated, and genetic deletion of p16^INK4a^ partially rescued the phenotype [101]. Genetic deletion of the *INK4a* or *ARF* locus led to defects in the hyaloid vascular system and retinal dysplasia, as in human persistent hyperplastic primary vitreous, independently of p53 [102,103,104]. In avians, retina SA β-Gal activity was observed in photoreceptors and RPE during development [105]. In old human retina, p16^INK4a^ is expressed in rods, ganglion cells, amacrine cells, and horizontal cells. Moreover, p16^INK4a^ and p21^Cip1^ are expressed in retinal vascular vessels, elucidating the expression of canonical senescence markers in retinal cells [106].

**Figure 3:**
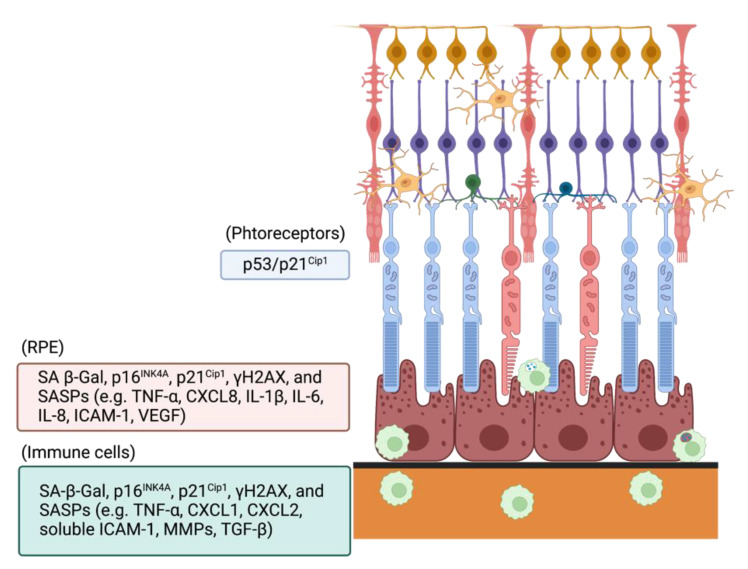
Oxidative stress-induced senescence markers associated with AMD in the retinal cells.

Based on the role of oxidative stress-induced cellular senescence in vitro [107], SIPS induced by oxidative stress is implicated in the pathogenesis of AMD (Figure 3). Senescence markers including SA β-Gal, p16^INK4a^, p21^Cip1^, and p53 are upregulated in animal AMD models [108,109]. Senescent RPE cells were observed around drusen in primates [110], and the expression of p16^INK4a^ increased in RPE from patients with geographic atrophy compared to age-matched controls [111]. Conversely, subretinal deposits morphologically separate and disturb RPE cells in AMD patients, indicating that drusen facilitates RPE dysfunction and senescence [112]. Oxidative stress drives senescence in RPE cells. Hydrogen peroxide and cigarette smoke increase the expression of SA β-Gal, p16^INK4a^, p21^Cip1^, γH2AX, and SASPs in ARPE-19 cells [113]. Hydrogen peroxide increased SA β-Gal activity and several senescence markers including apolipoprotein J, connective tissue growth factor, and fibronectin by upregulating TGF-β1 and TGF-β2 [114]. Mechanistically, hydrogen peroxide-induced senescence in RPE cells activates the p53/p21^Cip1^ pathway by upregulating bone morphogenetic protein-4 in the RPE layer and thickened Bruch membrane adjacent to drusen in retinal sections from early and late AMD patients [115]. Phagocytosis of oxidized products also induces senescence in RPE cells. Oxidized photoreceptor outer segments accelerate SIPS of RPE cells and the dysregulation of SASPs including TNF-α, IL-8, VEGF, and CFH [113,116]. Westlund et al. reported that ARPE-19 cells loading photooxidized A2E by blue light exhibited the cell death of RPE cells and SASP secretion. Apoptosis was upregulated by c-Abl and p53 and was suppressed by the inhibition of TP53 [117].

mTOR signaling is implicated in oxidative stress-induced senescence in RPE cells. Senescent RPE cells with high cumulative population doubling have increased sensitivity to mTORC1 signaling as a response to exogenous nutrient stimuli. Rapamycin suppressed senescence in RPE cells by inhibiting SA-β-Gal activity and the expression of p16^INK4a^ [118]. Activation of autophagy by rapamycin repressed SIPS, indicating that rapamycin suppresses oxidative stress-induced senescence in RPE cells.

Global genetic deletion of Nrf2 and peroxisome proliferator-activated receptor gamma coactivator-1α triggered RPE degeneration associated with increased endoplasmic reticulum stress in RPE cells and thickened Bruch’s membrane. The autophagy markers p62/SQSTM1 and LC3B, and the oxidative stress marker 4-HNE (4-hydroxynonenal) were upregulated in this mice model [119]. Nrf2 regulates autophagy and antioxidant responses and mediates anti-inflammatory effects in RPE cells by interacting with p62 [120]. Inhibition of the synthesis of glutathione, an antioxidant downstream of Nrf2, in ARPE-19 cells arrested the cell cycle at the G_1_ phase, and increased SA-β-Gal activity and SASPs including IL-6 and IL-8 [121]. These findings suggest that Nrf2 suppresses AMD by inhibiting oxidative stress-induced senescence. Several antioxidants exert beneficial effects against cellular senescence via Nrf2 signaling [122]. Lutein repressed hydrogen peroxide-induced ROS production and decreased SA-β-Gal activity in ARPE-19 cells, in part by upregulating sirtuin (SIRT)-1, and SIRT3. Therefore, the effect of lutein on AMD progression is mediated by its inhibition of oxidative stress and senescence [123].

Although functional and morphological impairment of photoreceptors is a sign of AMD, there are few reports of cellular senescence in photoreceptors. Miller et al. demonstrated that a radiosensitizing anticancer agent CI-1010, (R)-alpha-[[(2-bromoethyl)amino]methyl]-2-nitro-1H-imidazole-1-ethanol monohydrobromide, which induces oxidative stress, caused apoptosis of 661 W cells (an immortalized cone photoreceptor cell line derived from the retinal tumor of a mouse) by activating caspase-3 [124]. Cell death is reportedly caused by increased expression and phosphorylation of p53 in 661W cells. Therefore, oxidative stress triggers photoreceptor senescence and results in apoptosis.

The innate immune system is crucial in homeostasis maintenance and senescent cell clearance. Senescent cells activate and are cleared by NK cells, monocytes/macrophages, and T cells in multiple tissues [125]. In other words, immune cells eliminate senescent cells in a healthy state. However, the age-related decline in innate immune cell function has deleterious effects, and failure to clear senescent cells leads to their accumulation, aggravating senescence. Therefore, the age-related dysregulation and functional decline of immune cells accelerates aging and age-related diseases. In terms of the innate immune system, the alteration in biological functions declines with age, along with the accumulation of senescent cells, and it leads to angiogenic and inflammatory response [126]. Senescent and young macrophages enhanced and suppressed, respectively, choroidal neovascularization in a mouse model [127]. As such, the dysregulation of immune activation with age is a driver of AMD [128]. Aged retinal microglia show increased expression of C3 and complement factors, which are risk factors for AMD [129]. Immunosenescence, also termed immune-cell senescence, may be associated with the development of AMD.

Hydrogen peroxide induces the production of γH2AX in macrophages [130]. γ-Radiation increases oxidative stress and the expression of p16^INK4a^ and p21^Cip1^, SA-β-Gal, and SASPs (CXCL1, CXCL2, TNF-α, and soluble ICAM-1) in macrophages in vitro, which was suppressed by resolvin D1 [131]. Drusen components induce the production of IL-1β and IL-18 in human peripheral blood mononuclear cells, partially by activating the NLRP3 inflammasome [132]. Therefore, oxidative stress in AMD accelerates immunosenescence and inflammation. Additionally, aging facilitates cellular senescence and lipofuscin accumulation in immune cells [133]. Senescent microglia and/or macrophages migrate to the subretinal space between the neurosensory retina and RPE cells with age. These cells contain lipofuscin, implicating senescent immune cells in the aging retina [134].

In vivo, intravitreal injection of iron induces photoreceptor death and lipofuscin accumulation in RPE cells, mimicking geographic atrophy [135]. Liu et al. found that intravitreal injection of ferric ammonium citrate induced the lipofuscin formation in the outer segment of photoreceptors, RPE cells, and subretinal myeloid cells. Increased 8-hydroxy-2′-deoxyguanosine (8-OHdG), a DNA oxidation product, was found in RPE cells. Carboxyethyl pyrrole was initially found in the photoreceptors and accumulated in RPE cells and subretinal myeloid cells [136]. Malondialdehyde and oxidized phospholipids were present in RPE cells and subretinal myeloid cells, suggesting that oxidative stress and lipid peroxidation underlie lipofuscinogenesis and cellular senescence in an AMD model.

Cellular senescence is also associated with oxygen-induced retinopathy (OIR) as an animal model of proliferative diabetic retinopathy. Senescent cells accumulate in retina human proliferative diabetic retinopathy. In the OIR model, the retina expresses increased senescent markers (p16^INK4a^ p21^Cip1^, and SA-β-Gal) and SASPs including VEGF, IL-1β, IL-6, TGFβ-1, and plasminogen activator inhibitor 1 (Pai1) [137]. Cells constituting vascular units such as vascular endothelial cells, pericytes, astrocytes, and Müller glia were particularly increased transcripts associated with cellular senescence [138], which suggested the presence of senescent cells in neovascular tufts [139]. 

In terms of glaucoma as another age-related ocular disease, a genome-wide association study found that p16^INK4a^ loci is a strong risk factor gene for human primary open angle glaucoma (POAG) [140]. Increased senescent cells are observed in the outflow pathway and retinal ganglion cells (RGCs) in human glaucoma eyes, and the elevation of intraocular pressure induces their senescence [141,142]. p16^INK4a^ induce RGC senescence and death as a downstream of SIX6 [142]. Additionally, human eyes with acute primary angle-closure express increased ROS, 8-OHdG, malondialdehyde, and SASPs such as IL-6, IL-8, TNF-α, CCL2, GROα, MIP-1α, VEGF, IGFBP5, IGFBP7, and TGF-β 1 in aqueous humor. Among them, IL-6, IL-8, CCL2, GROα, MIP-1α, IGFBP5, IGFBP7, and TGF-β 1 significantly correlated with ROS, indicating that oxidative stress and cellular senescence are associated with the pathology in cooperation with one another [143]. 

## 5. Senolytics Targeting Oxidative Stress-Induced Cellular Senescence

In several animal models, investigations to examine curative effects against AMD targeting oxidative stress and cellular senescence have been performed. Several senolytic strategies have been established in vivo and in vitro, based on selective elimination of senescent cells by genetic means (INK-ATTAC, p16-3MR) [106,144], senolytic drugs [145], or inhibiting SASPs to suppress chronic inflammation. 

Senolysis suppresses OIR. The clearance of senescent cells facilitates healthy reparative vascular remodeling, indicating that senescent vascular endothelial cells have detrimental effects on pathological retinal angiogenesis. INK-ATTAC and a B-cell lymphoma-extra-large (BCL-xL) inhibitor suppressed OIR in mice, suggesting senolysis to be a therapeutic target in age-related retinal vascular diseases [138]. In fact, the B cell lymphoma-2 (BCL-2) family (BCL-W, BCL-XL, and BCL-2) promotes the resistance of senescent cells to apoptosis [146,147]. The BCL-2 family has potential as a therapeutic target in AMD, and a phase 2 clinical trial of the safety and efficacy of intravitreal injection of a BCL-xL inhibitor in patients with neovascular AMD is ongoing [148]. Regarding glaucoma, the clearance of senescent cells by p16-3MR transgenic mice, which show selective elimination of p16^INK4a^-positive senescent cells, preserved the number of living retinal ganglion cells and visual function in the presence of ocular hypertension [149]. Therefore, the clearance of senescent cells by senolysis suppresses age-related ocular diseases.

Quercetin is a bioflavonoid with senolytic and anti-inflammatory effects. Quercetin suppresses inflammatory cytokines via NF-κB signaling and inhibits cytotoxicity induced by hydrogen peroxide by upregulating Nrf2 and its downstream factors in ARPE-19 cells [150,151]. Quercetin also suppressed cigarette smoke extract-induced apoptosis and the expression of IL-1 β, IL-16, and IL-8, and upregulated Nrf2 in ARPE-19 cells [152], suggesting that quercetin inhibits oxidative stress-induced senescence. The senolytic drug dasatinib suppresses choroidal neovascularization, OIR, and retinal fibrosis [153,154]. Metformin, an AMPK activator, also has an anti-senescence effect [79]. Because metformin decreases the risk of AMD [155], it may have a preventive effect on AMD. In vitro, metformin enhanced ARPE-19 cell viability under oxidative stress, reduced ROS production, and increased Sirt1 and Nrf2 expression [156]. Mechanistically, metformin attenuated hydrogen peroxide-induced cell death, ROS production, and the collapse of the mitochondrial membrane potential in RPE cells by activating autophagy [157]. Metformin also suppressed pathological angiogenesis in an OIR model [137].

Fatty acids and their metabolites have antioxidant effects and suppress the progression of AMD [158]. In AMD, amyloid-β accumulates as a component of drusen [159]. Amyloid-β has a close molecular biological interaction with oxidative stress in a feedback loop [160]. Do et al. reported that elovanoids, which are synthesized from omega-3 very long chain polyunsaturated fatty acids, repressed the oligomeric β-amyloid-induced pathological upregulation of senescence markers (p16^INK4a^, p21 ^Cip1^, and p53), SASP (IL-1β, VEGF, and MMPs), and autophagy in RPE cells, and suppressed apoptosis genes in the neurosensory retina [161].

## 6. Conclusions and Future Perspectives

Oxidative stress triggers cellular senescence, driving age-related diseases. The accumulation of senescent retinal cells leads to AMD. Although oxidative stress-induced cellular senescence is implicated in the pathogenesis of AMD, the mechanism is unclear. Further studies should focus on the mechanistic aspects to elucidate the translational implications. 

## References

[1] Flatt T. (2012). A new definition of aging?. Front. Genet..

[2] Ni Y.Q., Liu Y.S. (2021). New Insights into the Roles and Mechanisms of Spermidine in Aging and Age-Related Diseases. Aging Dis..

[3] Wong T.Y., Chakravarthy U., Klein R., Mitchell P., Zlateva G., Buggage R., Fahrbach K., Probst C., Sledge I. (2008). The natural history and prognosis of neovascular age-related macular degeneration: A systematic review of the literature and meta-analysis. Ophthalmology.

[4] Wong W.L., Su X., Li X., Cheung C.M., Klein R., Cheng C.Y., Wong T.Y. (2014). Global prevalence of age-related macular degeneration and disease burden projection for 2020 and 2040: A systematic review and meta-analysis. Lancet Glob. Health.

[5] Sarks S.H. (1976). Ageing and degeneration in the macular region: A clinico-pathological study. Br. J. Ophthalmol..

[6] Apte R.S. (2021). Age-Related Macular Degeneration. N. Engl. J. Med..

[7] Lim L.S., Mitchell P., Seddon J.M., Holz F.G., Wong T.Y. (2012). Age-related macular degeneration. Lancet.

[8] Elfawy H.A., Das B. (2019). Crosstalk between mitochondrial dysfunction, oxidative stress, and age related neurodegenerative disease: Etiologies and therapeutic strategies. Life Sci..

[9] Luo J., Mills K., le Cessie S., Noordam R., van Heemst D. (2020). Ageing, age-related diseases and oxidative stress: What to do next?. Ageing Res. Rev..

[10] Zhang Z.Y., Bao X.L., Cong Y.Y., Fan B., Li G.Y. (2020). Autophagy in Age-Related Macular Degeneration: A Regulatory Mechanism of Oxidative Stress. Oxid. Med. Cell. Longev..

[11] Chen Q., Fischer A., Reagan J.D., Yan L.J., Ames B.N. (1995). Oxidative DNA damage and senescence of human diploid fibroblast cells. Proc. Natl. Acad. Sci. USA.

[12] Munoz-Espin D., Serrano M. (2014). Cellular senescence: From physiology to pathology. Nat. Rev. Mol. Cell Biol..

[13] Caceres-Velez P.R., Hui F., Hercus J., Bui B., Jusuf P.R. (2022). Restoring the oxidative balance in age-related diseases—An approach in glaucoma. Ageing Res. Rev..

[14] Moldogazieva N.T., Mokhosoev I.M., Mel’nikova T.I., Porozov Y.B., Terentiev A.A. (2019). Oxidative Stress and Advanced Lipoxidation and Glycation End Products (ALEs and AGEs) in Aging and Age-Related Diseases. Oxid. Med. Cell. Longev..

[15] Brand M.D., Orr A.L., Perevoshchikova I.V., Quinlan C.L. (2013). The role of mitochondrial function and cellular bioenergetics in ageing and disease. Br. J. Dermatol..

[16] Kauppinen A., Paterno J.J., Blasiak J., Salminen A., Kaarniranta K. (2016). Inflammation and its role in age-related macular degeneration. Cell. Mol. Life Sci..

[17] Neofytou E., Tzortzaki E.G., Chatziantoniou A., Siafakas N.M. (2012). DNA damage due to oxidative stress in Chronic Obstructive Pulmonary Disease (COPD). Int. J Mol. Sci..

[18] Zhao Z., Dong Q., Liu X., Wei L., Liu L., Li Y., Wang X. (2020). Dynamic transcriptome profiling in DNA damage-induced cellular senescence and transient cell-cycle arrest. Genomics.

[19] Aiken C.T., Kaake R.M., Wang X., Huang L. (2011). Oxidative stress-mediated regulation of proteasome complexes. Mol. Cell. Proteom..

[20] Jarrett S.G., Lin H., Godley B.F., Boulton M.E. (2008). Mitochondrial DNA damage and its potential role in retinal degeneration. Prog. Retin. Eye Res..

[21] Balistreri C.R., Candore G., Accardi G., Colonna-Romano G., Lio D. (2013). NF-kappaB pathway activators as potential ageing biomarkers: Targets for new therapeutic strategies. Immun. Ageing.

[22] Lin M.M., Liu N., Qin Z.H., Wang Y. (2022). Mitochondrial-derived damage-associated molecular patterns amplify neuroinflammation in neurodegenerative diseases. Acta Pharmacol. Sin..

[23] Liu T., Zhang L., Joo D., Sun S.C. (2017). NF-kappaB signaling in inflammation. Signal Transduct. Target. Ther..

[24] Lingappan K. (2018). NF-kappaB in Oxidative Stress. Curr. Opin. Toxicol..

[25] Yu C., Xiao J.H. (2021). The Keap1-Nrf2 System: A Mediator between Oxidative Stress and Aging. Oxid. Med. Cell. Longev..

[26] Hellyer J.A., Padda S.K., Diehn M., Wakelee H.A. (2021). Clinical Implications of KEAP1-NFE2L2 Mutations in NSCLC. J. Thorac. Oncol..

[27] Sajadimajd S., Khazaei M. (2018). Oxidative Stress and Cancer: The Role of Nrf2. Curr. Cancer Drug Targets.

[28] Bellezza I., Giambanco I., Minelli A., Donato R. (2018). Nrf2-Keap1 signaling in oxidative and reductive stress. Biochim. Biophys. Acta Mol. Cell Res..

[29] Lee K.S., Lin S., Copland D.A., Dick A.D., Liu J. (2021). Cellular senescence in the aging retina and developments of senotherapies for age-related macular degeneration. J. Neuroinflamm..

[30] Mitter S.K., Song C., Qi X., Mao H., Rao H., Akin D., Lewin A., Grant M., Dunn W., Ding J. (2014). Dysregulated autophagy in the RPE is associated with increased susceptibility to oxidative stress and AMD. Autophagy.

[31] Kaarniranta K., Tokarz P., Koskela A., Paterno J., Blasiak J. (2017). Autophagy regulates death of retinal pigment epithelium cells in age-related macular degeneration. Cell Biol. Toxicol..

[32] Ballinger S.W., Van Houten B., Jin G.F., Conklin C.A., Godley B.F. (1999). Hydrogen peroxide causes significant mitochondrial DNA damage in human RPE cells. Exp. Eye Res..

[33] Jin G.F., Hurst J.S., Godley B.F. (2001). Hydrogen peroxide stimulates apoptosis in cultured human retinal pigment epithelial cells. Curr. Eye Res..

[34] Hanus J., Zhang H., Wang Z., Liu Q., Zhou Q., Wang S. (2013). Induction of necrotic cell death by oxidative stress in retinal pigment epithelial cells. Cell Death Dis..

[35] Brown E.E., DeWeerd A.J., Ildefonso C.J., Lewin A.S., Ash J.D. (2019). Mitochondrial oxidative stress in the retinal pigment epithelium (RPE) led to metabolic dysfunction in both the RPE and retinal photoreceptors. Redox Biol..

[36] Piippo N., Korhonen E., Hytti M., Kinnunen K., Kaarniranta K., Kauppinen A. (2018). Oxidative Stress is the Principal Contributor to Inflammasome Activation in Retinal Pigment Epithelium Cells with Defunct Proteasomes and Autophagy. Cell. Physiol. Biochem..

[37] Wang K., Yao Y., Zhu X., Zhang K., Zhou F., Zhu L. (2017). Amyloid beta induces NLRP3 inflammasome activation in retinal pigment epithelial cells via NADPH oxidase- and mitochondria-dependent ROS production. J. Biochem. Mol. Toxicol..

[38] Blevins H.M., Xu Y., Biby S., Zhang S. (2022). The NLRP3 Inflammasome Pathway: A Review of Mechanisms and Inhibitors for the Treatment of Inflammatory Diseases. Front. Aging Neurosci..

[39] Ruan Y., Jiang S., Gericke A. (2021). Age-Related Macular Degeneration: Role of Oxidative Stress and Blood Vessels. Int. J. Mol. Sci.

[40] Kim Y.W., Byzova T.V. (2014). Oxidative stress in angiogenesis and vascular disease. Blood.

[41] Monaghan-Benson E., Hartmann J., Vendrov A.E., Budd S., Byfield G., Parker A., Ahmad F., Huang W., Runge M., Burridge K. (2010). The role of vascular endothelial growth factor-induced activation of NADPH oxidase in choroidal endothelial cells and choroidal neovascularization. Am. J. Pathol..

[42] Collado M., Blasco M.A., Serrano M. (2007). Cellular senescence in cancer and aging. Cell.

[43] Di Micco R., Krizhanovsky V., Baker D., d’Adda di Fagagna F. (2021). Cellular senescence in ageing: From mechanisms to therapeutic opportunities. Nat. Rev. Mol. Cell Biol..

[44] Childs B.G., Durik M., Baker D.J., van Deursen J.M. (2015). Cellular senescence in aging and age-related disease: From mechanisms to therapy. Nat. Med..

[45] Kumari R., Jat P. (2021). Mechanisms of Cellular Senescence: Cell Cycle Arrest and Senescence Associated Secretory Phenotype. Front. Cell Dev. Biol..

[46] Terman A., Kurz T., Navratil M., Arriaga E.A., Brunk U.T. (2010). Mitochondrial turnover and aging of long-lived postmitotic cells: The mitochondrial-lysosomal axis theory of aging. Antioxid. Redox Signal..

[47] Moreno-Garcia A., Kun A., Calero O., Medina M., Calero M. (2018). An Overview of the Role of Lipofuscin in Age-Related Neurodegeneration. Front. Neurosci..

[48] Rizou S.V., Evangelou K., Myrianthopoulos V., Mourouzis I., Havaki S., Athanasiou A., Vasileiou P.V.S., Margetis A., Kotsinas A., Kastrinakis N.G. (2019). A Novel Quantitative Method for the Detection of Lipofuscin, the Main By-Product of Cellular Senescence, in Fluids. Methods Mol. Biol..

[49] Georgakopoulou E.A., Tsimaratou K., Evangelou K., Fernandez Marcos P.J., Zoumpourlis V., Trougakos I.P., Kletsas D., Bartek J., Serrano M., Gorgoulis V.G. (2013). Specific lipofuscin staining as a novel biomarker to detect replicative and stress-induced senescence. A method applicable in cryo-preserved and archival tissues. Aging (Albany NY).

[50] Salmonowicz H., Passos J.F. (2017). Detecting senescence: A new method for an old pigment. Aging Cell.

[51] Goulielmaki E., Ioannidou A., Tsekrekou M., Stratigi K., Poutakidou I.K., Gkirtzimanaki K., Aivaliotis M., Evangelou K., Topalis P., Altmuller J. (2020). Tissue-infiltrating macrophages mediate an exosome-based metabolic reprogramming upon DNA damage. Nat. Commun..

[52] Konig J., Ott C., Hugo M., Jung T., Bulteau A.L., Grune T., Hohn A. (2017). Mitochondrial contribution to lipofuscin formation. Redox Biol..

[53] Vida C., de Toda I.M., Cruces J., Garrido A., Gonzalez-Sanchez M., De la Fuente M. (2017). Role of macrophages in age-related oxidative stress and lipofuscin accumulation in mice. Redox Biol..

[54] von Kobbe C. (2018). Cellular senescence: A view throughout organismal life. Cell. Mol. Life Sci..

[55] Campisi J. (1997). The biology of replicative senescence. Eur. J. Cancer.

[56] Beck J., Horikawa I., Harris C. (2020). Cellular Senescence: Mechanisms, Morphology, and Mouse Models. Vet. Pathol..

[57] Munoz-Espin D., Canamero M., Maraver A., Gomez-Lopez G., Contreras J., Murillo-Cuesta S., Rodriguez-Baeza A., Varela-Nieto I., Ruberte J., Collado M. (2013). Programmed cell senescence during mammalian embryonic development. Cell.

[58] Macip S., Igarashi M., Fang L., Chen A., Pan Z.Q., Lee S.W., Aaronson S.A. (2002). Inhibition of p21-mediated ROS accumulation can rescue p21-induced senescence. EMBO J..

[59] Chen J., Goligorsky M.S. (2006). Premature senescence of endothelial cells: Methusaleh’s dilemma. Am. J. Physiol. Heart Circ. Physiol..

[60] Song Y.S., Lee B.Y., Hwang E.S. (2005). Dinstinct ROS and biochemical profiles in cells undergoing DNA damage-induced senescence and apoptosis. Mech. Ageing Dev..

[61] McConnell B.B., Gregory F.J., Stott F.J., Hara E., Peters G. (1999). Induced expression of p16(INK4a) inhibits both CDK4- and CDK2-associated kinase activity by reassortment of cyclin-CDK-inhibitor complexes. Mol. Cell. Biol..

[62] Xue W., Zender L., Miething C., Dickins R.A., Hernando E., Krizhanovsky V., Cordon-Cardo C., Lowe S.W. (2007). Senescence and tumour clearance is triggered by p53 restoration in murine liver carcinomas. Nature.

[63] Liu Y., Sharpless N.E. (2009). Tumor suppressor mechanisms in immune aging. Curr. Opin. Immunol..

[64] Quesnel B. (1998). [Inhibitors of cyclins/CDK of the 9p21 chromosomal region and malignant hemopathies]. Bull. Cancer.

[65] Romagosa C., Simonetti S., Lopez-Vicente L., Mazo A., Lleonart M.E., Castellvi J., Ramon y Cajal S. (2011). p16(Ink4a) overexpression in cancer: A tumor suppressor gene associated with senescence and high-grade tumors. Oncogene.

[66] Zindy F., Quelle D.E., Roussel M.F., Sherr C.J. (1997). Expression of the p16INK4a tumor suppressor versus other INK4 family members during mouse development and aging. Oncogene.

[67] Krishnamurthy J., Torrice C., Ramsey M.R., Kovalev G.I., Al-Regaiey K., Su L., Sharpless N.E. (2004). Ink4a/Arf expression is a biomarker of aging. J. Clin. Invest..

[68] Baker D.J., Childs B.G., Durik M., Wijers M.E., Sieben C.J., Zhong J., Saltness R.A., Jeganathan K.B., Verzosa G.C., Pezeshki A. (2016). Naturally occurring p16(Ink4a)-positive cells shorten healthy lifespan. Nature.

[69] Jung Y.S., Qian Y., Chen X. (2010). Examination of the expanding pathways for the regulation of p21 expression and activity. Cell Signal..

[70] Al Bitar S., Gali-Muhtasib H. (2019). The Role of the Cyclin Dependent Kinase Inhibitor p21(cip1/waf1) in Targeting Cancer: Molecular Mechanisms and Novel Therapeutics. Cancers.

[71] Yosef R., Pilpel N., Papismadov N., Gal H., Ovadya Y., Vadai E., Miller S., Porat Z., Ben-Dor S., Krizhanovsky V. (2017). p21 maintains senescent cell viability under persistent DNA damage response by restraining JNK and caspase signaling. EMBO J..

[72] Karimian A., Ahmadi Y., Yousefi B. (2016). Multiple functions of p21 in cell cycle, apoptosis and transcriptional regulation after DNA damage. DNA Repair (Amst).

[73] Lee B.Y., Han J.A., Im J.S., Morrone A., Johung K., Goodwin E.C., Kleijer W.J., DiMaio D., Hwang E.S. (2006). Senescence-associated beta-galactosidase is lysosomal beta-galactosidase. Aging Cell.

[74] Katsuumi G., Shimizu I., Yoshida Y., Minamino T. (2018). Vascular Senescence in Cardiovascular and Metabolic Diseases. Front. Cardiovasc. Med..

[75] Ksiazek K., Piwocka K., Brzezinska A., Sikora E., Zabel M., Breborowicz A., Jorres A., Witowski J. (2006). Early loss of proliferative potential of human peritoneal mesothelial cells in culture: The role of p16INK4a-mediated premature senescence. J. Appl. Physiol..

[76] Patil P., Falabella M., Saeed A., Lee D., Kaufman B., Shiva S., Croix C.S., Van Houten B., Niedernhofer L.J., Robbins P.D. (2019). Oxidative stress-induced senescence markedly increases disc cell bioenergetics. Mech. Ageing Dev..

[77] Ok C.Y., Park S., Jang H.O., Takata T., Lee O.H., Bae M.K., Bae S.K. (2021). FK866 Protects Human Dental Pulp Cells against Oxidative Stress-Induced Cellular Senescence. Antioxidants.

[78] Faget D.V., Ren Q., Stewart S.A. (2019). Unmasking senescence: Context-dependent effects of SASP in cancer. Nat. Rev. Cancer.

[79] Han X., Zhang T., Zhang X., Zhang R., Lao K., Mi Y., Gou X. (2020). AMPK alleviates oxidative stressinduced premature senescence via inhibition of NF-kappaB/STAT3 axis-mediated positive feedback loop. Mech. Ageing Dev..

[80] Salminen A., Kauppinen A., Kaarniranta K. (2012). Emerging role of NF-kappaB signaling in the induction of senescence-associated secretory phenotype (SASP). Cell Signal..

[81] Chien Y., Scuoppo C., Wang X., Fang X., Balgley B., Bolden J.E., Premsrirut P., Luo W., Chicas A., Lee C.S. (2011). Control of the senescence-associated secretory phenotype by NF-kappaB promotes senescence and enhances chemosensitivity. Genes Dev..

[82] Rovillain E., Mansfield L., Caetano C., Alvarez-Fernandez M., Caballero O.L., Medema R.H., Hummerich H., Jat P.S. (2011). Activation of nuclear factor-kappa B signalling promotes cellular senescence. Oncogene.

[83] Zhang H., Davies K.J.A., Forman H.J. (2015). Oxidative stress response and Nrf2 signaling in aging. Free Radic Biol. Med..

[84] Suh J.H., Shenvi S.V., Dixon B.M., Liu H., Jaiswal A.K., Liu R.M., Hagen T.M. (2004). Decline in transcriptional activity of Nrf2 causes age-related loss of glutathione synthesis, which is reversible with lipoic acid. Proc. Natl. Acad. Sci. USA.

[85] Pomatto L.C.D., Dill T., Carboneau B., Levan S., Kato J., Mercken E.M., Pearson K.J., Bernier M., de Cabo R. (2020). Deletion of Nrf2 shortens lifespan in C57BL6/J male mice but does not alter the health and survival benefits of caloric restriction. Free Radic. Biol. Med..

[86] Gorbunova V., Rezazadeh S., Seluanov A. (2016). Dangerous Entrapment for NRF2. Cell.

[87] Kapeta S., Chondrogianni N., Gonos E.S. (2010). Nuclear erythroid factor 2-mediated proteasome activation delays senescence in human fibroblasts. J. Biol. Chem..

[88] Wati S.M., Matsumaru D., Motohashi H. (2020). NRF2 pathway activation by KEAP1 inhibition attenuates the manifestation of aging phenotypes in salivary glands. Redox Biol..

[89] Fulop G.A., Kiss T., Tarantini S., Balasubramanian P., Yabluchanskiy A., Farkas E., Bari F., Ungvari Z., Csiszar A. (2018). Nrf2 deficiency in aged mice exacerbates cellular senescence promoting cerebrovascular inflammation. Geroscience.

[90] Chen W., Sun Z., Wang X.J., Jiang T., Huang Z., Fang D., Zhang D.D. (2009). Direct interaction between Nrf2 and p21(Cip1/WAF1) upregulates the Nrf2-mediated antioxidant response. Mol. Cell.

[91] Nakano-Kobayashi A., Fukumoto A., Morizane A., Nguyen D.T., Le T.M., Hashida K., Hosoya T., Takahashi R., Takahashi J., Hori O. (2020). Therapeutics potentiating microglial p21-Nrf2 axis can rescue neurodegeneration caused by neuroinflammation. Sci. Adv..

[92] Matsumaru D., Motohashi H. (2021). The KEAP1-NRF2 System in Healthy Aging and Longevity. Antioxidants.

[93] Powell J.D., Pollizzi K.N., Heikamp E.B., Horton M.R. (2012). Regulation of immune responses by mTOR. Annu. Rev. Immunol..

[94] Weichhart T. (2018). mTOR as Regulator of Lifespan, Aging, and Cellular Senescence: A Mini-Review. Gerontology.

[95] Ehninger D., Neff F., Xie K. (2014). Longevity, aging and rapamycin. Cell. Mol. Life Sci..

[96] Xue Q.L., Yang H., Li H.F., Abadir P.M., Burks T.N., Koch L.G., Britton S.L., Carlson J., Chen L., Walston J.D. (2016). Rapamycin increases grip strength and attenuates age-related decline in maximal running distance in old low capacity runner rats. Aging (Albany NY).

[97] Laberge R.M., Sun Y., Orjalo A.V., Patil C.K., Freund A., Zhou L., Curran S.C., Davalos A.R., Wilson-Edell K.A., Liu S. (2015). MTOR regulates the pro-tumorigenic senescence-associated secretory phenotype by promoting IL1A translation. Nat. Cell Biol..

[98] Mannick J.B., Del Giudice G., Lattanzi M., Valiante N.M., Praestgaard J., Huang B., Lonetto M.A., Maecker H.T., Kovarik J., Carson S. (2014). mTOR inhibition improves immune function in the elderly. Sci. Transl. Med..

[99] Wang R., Yu Z., Sunchu B., Shoaf J., Dang I., Zhao S., Caples K., Bradley L., Beaver L.M., Ho E. (2017). Rapamycin inhibits the secretory phenotype of senescent cells by a Nrf2-independent mechanism. Aging Cell.

[100] Wagner K.D., Wagner N. (2022). The Senescence Markers p16INK4A, p14ARF/p19ARF, and p21 in Organ Development and Homeostasis. Cells.

[101] Takeda S., Sasagawa S., Oyama T., Searleman A.C., Westergard T.D., Cheng E.H., Hsieh J.J. (2015). Taspase1-dependent TFIIA cleavage coordinates head morphogenesis by limiting Cdkn2a locus transcription. J. Clin. Invest..

[102] Cheong C., Sung Y.H., Lee J., Choi Y.S., Song J., Kee C., Lee H.W. (2006). Role of INK4a locus in normal eye development and cataract genesis. Mech. Ageing Dev..

[103] McKeller R.N., Fowler J.L., Cunningham J.J., Warner N., Smeyne R.J., Zindy F., Skapek S.X. (2002). The Arf tumor suppressor gene promotes hyaloid vascular regression during mouse eye development. Proc. Natl. Acad. Sci. USA.

[104] Silva R.L., Thornton J.D., Martin A.C., Rehg J.E., Bertwistle D., Zindy F., Skapek S.X. (2005). Arf-dependent regulation of Pdgf signaling in perivascular cells in the developing mouse eye. EMBO J..

[105] de Mera-Rodriguez J.A., Alvarez-Hernan G., Ganan Y., Martin-Partido G., Rodriguez-Leon J., Francisco-Morcillo J. (2019). Senescence-associated beta-galactosidase activity in the developing avian retina. Dev. Dyn..

[106] Lopez-Luppo M., Catita J., Ramos D., Navarro M., Carretero A., Mendes-Jorge L., Munoz-Canoves P., Rodriguez-Baeza A., Nacher V., Ruberte J. (2017). Cellular Senescence Is Associated With Human Retinal Microaneurysm Formation During Aging. Invest. Ophthalmol. Vis. Sci..

[107] Blasiak J. (2020). Senescence in the pathogenesis of age-related macular degeneration. Cell. Mol. Life Sci..

[108] Vuong L., Conley S.M., Al-Ubaidi M.R. (2012). Expression and role of p53 in the retina. Invest. Ophthalmol. Vis. Sci..

[109] Sreekumar P.G., Reddy S.T., Hinton D.R., Kannan R. (2022). Mechanisms of RPE senescence and potential role of alphaB crystallin peptide as a senolytic agent in experimental AMD. Exp. Eye Res..

[110] Mishima K., Handa J.T., Aotaki-Keen A., Lutty G.A., Morse L.S., Hjelmeland L.M. (1999). Senescence-associated beta-galactosidase histochemistry for the primate eye. Invest. Ophthalmol. Vis. Sci..

[111] Shimizu H., Yamada K., Suzumura A., Kataoka K., Takayama K., Sugimoto M., Terasaki H., Kaneko H. (2020). Caveolin-1 Promotes Cellular Senescence in Exchange for Blocking Subretinal Fibrosis in Age-Related Macular Degeneration. Invest. Ophthalmol. Vis. Sci..

[112] Ach T., Tolstik E., Messinger J.D., Zarubina A.V., Heintzmann R., Curcio C.A. (2015). Lipofuscin redistribution and loss accompanied by cytoskeletal stress in retinal pigment epithelium of eyes with age-related macular degeneration. Invest. Ophthalmol. Vis. Sci..

[113] Marazita M.C., Dugour A., Marquioni-Ramella M.D., Figueroa J.M., Suburo A.M. (2016). Oxidative stress-induced premature senescence dysregulates VEGF and CFH expression in retinal pigment epithelial cells: Implications for Age-related Macular Degeneration. Redox Biol..

[114] Yu A.L., Fuchshofer R., Kook D., Kampik A., Bloemendal H., Welge-Lussen U. (2009). Subtoxic oxidative stress induces senescence in retinal pigment epithelial cells via TGF-beta release. Invest. Ophthalmol. Vis. Sci..

[115] Zhu D., Wu J., Spee C., Ryan S.J., Hinton D.R. (2009). BMP4 mediates oxidative stress-induced retinal pigment epithelial cell senescence and is overexpressed in age-related macular degeneration. J. Biol. Chem..

[116] Chen M., Forrester J.V., Xu H. (2007). Synthesis of complement factor H by retinal pigment epithelial cells is down-regulated by oxidized photoreceptor outer segments. Exp. Eye Res..

[117] Westlund B.S., Cai B., Zhou J., Sparrow J.R. (2009). Involvement of c-Abl, p53 and the MAP kinase JNK in the cell death program initiated in A2E-laden ARPE-19 cells by exposure to blue light. Apoptosis.

[118] Chen Y., Wang J., Cai J., Sternberg P. (2010). Altered mTOR signaling in senescent retinal pigment epithelium. Invest. Ophthalmol. Vis. Sci..

[119] Felszeghy S., Viiri J., Paterno J.J., Hyttinen J.M.T., Koskela A., Chen M., Leinonen H., Tanila H., Kivinen N., Koistinen A. (2019). Loss of NRF-2 and PGC-1alpha genes leads to retinal pigment epithelium damage resembling dry age-related macular degeneration. Redox Biol..

[120] Wang S., Wang X., Cheng Y., Ouyang W., Sang X., Liu J., Su Y., Liu Y., Li C., Yang L. (2019). Autophagy Dysfunction, Cellular Senescence, and Abnormal Immune-Inflammatory Responses in AMD: From Mechanisms to Therapeutic Potential. Oxid. Med. Cell. Longev..

[121] Sun Y., Zheng Y., Wang C., Liu Y. (2018). Glutathione depletion induces ferroptosis, autophagy, and premature cell senescence in retinal pigment epithelial cells. Cell Death Dis..

[122] Shao Z., Wang B., Shi Y., Xie C., Huang C., Chen B., Zhang H., Zeng G., Liang H., Wu Y. (2021). Senolytic agent Quercetin ameliorates intervertebral disc degeneration via the Nrf2/NF-kappaB axis. Osteoarthr. Cartil..

[123] Chae S.Y., Park S.Y., Park G. (2018). Lutein protects human retinal pigment epithelial cells from oxidative stressinduced cellular senescence. Mol. Med. Rep..

[124] Miller T.J., Schneider R.J., Miller J.A., Martin B.P., Al-Ubaidi M.R., Agarwal N., Dethloff L.A., Philbert M.A. (2006). Photoreceptor cell apoptosis induced by the 2-nitroimidazole radiosensitizer, CI-1010, is mediated by p53-linked activation of caspase-3. Neurotoxicology.

[125] Sagiv A., Krizhanovsky V. (2013). Immunosurveillance of senescent cells: The bright side of the senescence program. Biogerontology.

[126] Song P., An J., Zou M.H. (2020). Immune Clearance of Senescent Cells to Combat Ageing and Chronic Diseases. Cells.

[127] Kelly J., Ali Khan A., Yin J., Ferguson T.A., Apte R.S. (2007). Senescence regulates macrophage activation and angiogenic fate at sites of tissue injury in mice. J. Clin. Invest..

[128] Ambati J., Atkinson J.P., Gelfand B.D. (2013). Immunology of age-related macular degeneration. Nat. Rev. Immunol..

[129] Ma W., Cojocaru R., Gotoh N., Gieser L., Villasmil R., Cogliati T., Swaroop A., Wong W.T. (2013). Gene expression changes in aging retinal microglia: Relationship to microglial support functions and regulation of activation. Neurobiol. Aging.

[130] Wang P., Geng J., Gao J., Zhao H., Li J., Shi Y., Yang B., Xiao C., Linghu Y., Sun X. (2019). Macrophage achieves self-protection against oxidative stress-induced ageing through the Mst-Nrf2 axis. Nat. Commun..

[131] Sadhu S., Decker C., Sansbury B.E., Marinello M., Seyfried A., Howard J., Mori M., Hosseini Z., Arunachalam T., Finn A.V. (2021). Radiation-Induced Macrophage Senescence Impairs Resolution Programs and Drives Cardiovascular Inflammation. J. Immunol..

[132] Doyle S.L., Campbell M., Ozaki E., Salomon R.G., Mori A., Kenna P.F., Farrar G.J., Kiang A.S., Humphries M.M., Lavelle E.C. (2012). NLRP3 has a protective role in age-related macular degeneration through the induction of IL-18 by drusen components. Nat. Med..

[133] Singh Kushwaha S., Patro N., Kumar Patro I. (2018). A Sequential Study of Age-Related Lipofuscin Accumulation in Hippocampus and Striate Cortex of Rats. Ann. Neurosci..

[134] Xu H., Chen M., Manivannan A., Lois N., Forrester J.V. (2008). Age-dependent accumulation of lipofuscin in perivascular and subretinal microglia in experimental mice. Aging Cell.

[135] Shu W., Baumann B.H., Song Y., Liu Y., Wu X., Dunaief J.L. (2020). Ferrous but not ferric iron sulfate kills photoreceptors and induces photoreceptor-dependent RPE autofluorescence. Redox Biol..

[136] Liu Y., Bell B.A., Song Y., Kim H.J., Sterling J.K., Kim B.J., Poli M., Guo M., Zhang K., Rao A. (2021). Intraocular iron injection induces oxidative stress followed by elements of geographic atrophy and sympathetic ophthalmia. Aging Cell.

[137] Oubaha M., Miloudi K., Dejda A., Guber V., Mawambo G., Germain M.A., Bourdel G., Popovic N., Rezende F.A., Kaufman R.J. (2016). Senescence-associated secretory phenotype contributes to pathological angiogenesis in retinopathy. Sci. Transl. Med..

[138] Crespo-Garcia S., Tsuruda P.R., Dejda A., Ryan R.D., Fournier F., Chaney S.Y., Pilon F., Dogan T., Cagnone G., Patel P. (2021). Pathological angiogenesis in retinopathy engages cellular senescence and is amenable to therapeutic elimination via BCL-xL inhibition. Cell Metab..

[139] Binet F., Cagnone G., Crespo-Garcia S., Hata M., Neault M., Dejda A., Wilson A.M., Buscarlet M., Mawambo G.T., Howard J.P. (2020). Neutrophil extracellular traps target senescent vasculature for tissue remodeling in retinopathy. Science.

[140] Osman W., Low S.K., Takahashi A., Kubo M., Nakamura Y. (2012). A genome-wide association study in the Japanese population confirms 9p21 and 14q23 as susceptibility loci for primary open angle glaucoma. Hum. Mol. Genet..

[141] Liton P.B., Challa P., Stinnett S., Luna C., Epstein D.L., Gonzalez P. (2005). Cellular senescence in the glaucomatous outflow pathway. Exp. Gerontol..

[142] Skowronska-Krawczyk D., Zhao L., Zhu J., Weinreb R.N., Cao G., Luo J., Flagg K., Patel S., Wen C., Krupa M. (2015). P16INK4a Upregulation Mediated by SIX6 Defines Retinal Ganglion Cell Pathogenesis in Glaucoma. Mol. Cell.

[143] Ye D., Xu Y., Shi Y., Ji J., Lu X., Chen H., Huang R., Lu P., Li Y., Cheng L. (2022). Occurrence of Oxidative Stress and Premature Senescence in the Anterior Segment of Acute Primary Angle-Closure Eyes. Invest. Ophthalmol. Vis. Sci..

[144] Jeon O.H., Kim C., Laberge R.M., Demaria M., Rathod S., Vasserot A.P., Chung J.W., Kim D.H., Poon Y., David N. (2017). Local clearance of senescent cells attenuates the development of post-traumatic osteoarthritis and creates a pro-regenerative environment. Nat. Med..

[145] Xu M., Pirtskhalava T., Farr J.N., Weigand B.M., Palmer A.K., Weivoda M.M., Inman C.L., Ogrodnik M.B., Hachfeld C.M., Fraser D.G. (2018). Senolytics improve physical function and increase lifespan in old age. Nat. Med..

[146] Chang J., Wang Y., Shao L., Laberge R.M., Demaria M., Campisi J., Janakiraman K., Sharpless N.E., Ding S., Feng W. (2016). Clearance of senescent cells by ABT263 rejuvenates aged hematopoietic stem cells in mice. Nat. Med..

[147] Yosef R., Pilpel N., Tokarsky-Amiel R., Biran A., Ovadya Y., Cohen S., Vadai E., Dassa L., Shahar E., Condiotti R. (2016). Directed elimination of senescent cells by in.nhibition of BCL-W and BCL-XL. Nat. Commun..

[148] Safety, Tolerability, and Efficacy Study of UBX1325 in Patients With Neovascular Age-Related Macular Degeneration (ENVISION). https://clinicaltrials.gov/ct2/show/NCT05275205?term=Safety%2C+Tolerability%2C+and+Efficacy+Study+of+UBX1325+in+Patients+With+Neovascular+Age-Related+Macular+Degeneration+%28ENVISION%29&draw=2&rank=1.

[149] Rocha L.R., Nguyen Huu V.A., Palomino La Torre C., Xu Q., Jabari M., Krawczyk M., Weinreb R.N., Skowronska-Krawczyk D. (2020). Early removal of senescent cells protects retinal ganglion cells loss in experimental ocular hypertension. Aging Cell.

[150] Cheng S.C., Huang W.C., JH S.P., Wu Y.H., Cheng C.Y. (2019). Quercetin Inhibits the Production of IL-1beta-Induced Inflammatory Cytokines and Chemokines in ARPE-19 Cells via the MAPK and NF-kappaB Signaling Pathways. Int. J. Mol. Sci..

[151] Weng S., Mao L., Gong Y., Sun T., Gu Q. (2017). Role of quercetin in protecting ARPE19 cells against H2O2induced injury via nuclear factor erythroid 2 like 2 pathway activation and endoplasmic reticulum stress inhibition. Mol. Med. Rep..

[152] Zhu Q., Liu M., He Y., Yang B. (2019). Quercetin protect cigarette smoke extracts induced inflammation and apoptosis in RPE cells. Artif. Cells Nanomed. Biotechnol..

[153] Ueda S., Nunn B.M., Chauhan R., McDonald K., Kaplan H.J., O’Toole M.G., Tamiya S. (2021). Sustained dasatinib treatment prevents early fibrotic changes following ocular trauma. Graefes Arch. Clin. Exp. Ophthalmol..

[154] Seo S., Suh W. (2017). Antiangiogenic effect of dasatinib in murine models of oxygen-induced retinopathy and laser-induced choroidal neovascularization. Mol. Vis..

[155] Blitzer A.L., Ham S.A., Colby K.A., Skondra D. (2021). Association of Metformin Use With Age-Related Macular Degeneration: A Case-Control Study. JAMA Ophthalmol..

[156] Qu S., Zhang C., Liu D., Wu J., Tian H., Lu L., Xu G.T., Liu F., Zhang J. (2020). Metformin Protects ARPE-19 Cells from Glyoxal-Induced Oxidative Stress. Oxid. Med. Cell. Longev..

[157] Zhao X., Liu L., Jiang Y., Silva M., Zhen X., Zheng W. (2020). Protective Effect of Metformin against Hydrogen Peroxide-Induced Oxidative Damage in Human Retinal Pigment Epith.h.helial (RPE) Cells by Enhancing Autophagy through Activation of AMPK Pathway. Oxid. Med. Cell. Longev..

[158] Suzumura A., Terao R., Kaneko H. (2020). Protective Effects and Molecular Signaling of n-3 Fatty Acids on Oxidative Stress and Inflammation in Retinal Diseases. Antioxidants.

[159] Dentchev T., Milam A.H., Lee V.M., Trojanowski J.Q., Dunaief J.L. (2003). Amyloid-beta is found in drusen from some age-related macular degeneration retinas, but not in drusen from normal retinas. Mol. Vis..

[160] Guglielmotto M., Giliberto L., Tamagno E., Tabaton M. (2010). Oxidative stress mediates the pathogenic effect of different Alzheimer’s disease risk factors. Front. Aging Neurosci..

[161] Do K.V., Kautzmann M.I., Jun B., Gordon W.C., Nshimiyimana R., Yang R., Petasis N.A., Bazan N.G. (2019). Elovanoids counteract oligomeric beta-amyloid-induced gene expression and protect photoreceptors. Proc. Natl. Acad. Sci. USA.

